# Deficiency of Macf1 in osterix expressing cells decreases bone formation by Bmp2/Smad/Runx2 pathway

**DOI:** 10.1111/jcmm.14729

**Published:** 2019-11-11

**Authors:** Wu‐Xia Qiu, Xiao‐Li Ma, Xiao Lin, Fan Zhao, Di‐Jie Li, Zhi‐Hao Chen, Ke‐Wen Zhang, Ru Zhang, Pai Wang, Yun‐Yun Xiao, Zhi‐Ping Miao, Kai Dang, Xiao‐Yang Wu, Ai‐Rong Qian

**Affiliations:** ^1^ Laboratory for Bone Metabolism Key Laboratory for Space Bioscience and Biotechnology School of Life Sciences Northwestern Polytechnical University Xi'an China; ^2^ Research Center for Special Medicine and Health Systems Engineering School of Life Sciences Northwestern Polytechnical University Xi'an China; ^3^ NPU‐UAB Joint Laboratory for Bone Metabolism School of Life Sciences Northwestern Polytechnical University Xi'an China; ^4^ Ben May Department for Cancer Research The University of Chicago Chicago IL USA

**Keywords:** Bmp2 pathway, bone formation, Macf1, primary osteoblasts

## Abstract

Microtubule actin cross‐linking factor 1 (Macf1) is a spectraplakin family member known to regulate cytoskeletal dynamics, cell migration, neuronal growth and cell signal transduction. We previously demonstrated that knockdown of Macf1 inhibited the differentiation of MC3T3‐E1 cell line. However, whether Macf1 could regulate bone formation in vivo is unclear. To study the function and mechanism of Macf1 in bone formation and osteogenic differentiation, we established osteoblast‐specific Osterix (Osx) promoter‐driven Macf1 conditional knockout mice (*Macf1^f/f^Osx*‐Cre). The *Macf1^f/f^Osx*‐Cre mice displayed delayed ossification and decreased bone mass. Morphological and mechanical studies showed deteriorated trabecular microarchitecture and impaired biomechanical strength of femur in *Macf1^f/f^Osx*‐Cre mice. In addition, the differentiation of primary osteoblasts isolated from calvaria was inhibited in *Macf1^f/f^Osx*‐Cre mice. Deficiency of Macf1 in primary osteoblasts inhibited the expression of osteogenic marker genes (Col1, Runx2 and Alp) and the number of mineralized nodules. Furthermore, deficiency of Macf1 attenuated Bmp2/Smad/Runx2 signalling in primary osteoblasts of *Macf1^f/f^Osx*‐Cre mice. Together, these results indicated that Macf1 plays a significant role in bone formation and osteoblast differentiation by regulating Bmp2/Smad/Runx2 pathway, suggesting that Macf1 might be a therapeutic target for bone disease.

## Graphical abstract

**Deficiency of Macf1 inhibits bone formation by attenuating Bmp2/Smad/Runx2 signalling in osteoblasts.** *Macf1^f^^/f^Osx‐Cre* mice showed decreased bone mass, deteriorated bone microarchitecture and impaired bone strength. We demonstrated that Macf1 affected differentiation of primary osteoblasts through Bmp2/Smad/Runx2 signalling pathway.

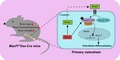

## 1 INTRODUCTION

Microtubule actin cross‐linking factor 1 (Macf1), also known as Actin cross‐linking factor 7 (Acf7), is a member of spectraplakin family.^1^, ^2^ Macf1 can crosslink with actin and microtubules and plays pivotal roles in cytoskeletal dynamics, cell migration, neuronal growth and cell signal transduction.^3^, ^4^, ^5^, ^6^ Studies have demonstrated that Macf1 has physiological functions in mammalian skin, nervous system, heart, retina, intestine and skeletal muscle.^6^, ^7^, ^8^, ^9^, ^10^, ^11^, ^12^, ^13^ However, the function of Macf1 in bone tissue remains unclear. In previous studies, Macf1 has been found to participate in the regulation of osteoblast differentiation‐associated Wnt/β‐catenin signalling pathway.^5^, ^6^, ^14^, ^15^ In our previous studies, it was demonstrated that Macf1 can regulate the proliferation, cell cycle progression and differentiation of MC3T3‐E1 osteoblastic cell line.^16^, ^17^, ^18^, ^19^ However, it remains unknown whether Macf1 could regulate bone formation in vivo.

The bone morphogenetic protein 2 (Bmp2) signalling pathway is a critical regulator of osteogenesis.^20^, ^21^ Bmp2 binds to its receptors to induce phosphorylation of Smad1/5/9. Activated Smads can form hetero complexes with Smad4, and then, the complexes are translocated into nucleus to regulate target genes such as runt‐related transcription factor 2 (Runx2) and osterix (Osx).^22^ Runx2 is a master transcription factor necessary for osteoblast differentiation, which can regulate the expression of osteoblast‐specific genes including alkaline phosphatase (Alp), collagen type I (Col1) and osteocalcin (Ocn).^23^ It has been reported that Wnt/β‐catenin pathway can regulate the activation of BMP2 transcription in osteoblasts.^5^, ^24^ Thus, we hypothesized that Macf1 could regulate osteoblast differentiation by modulating Bmp2 pathway.

To investigate the role of Macf1 in bone formation and osteoblast differentiation, we generated a mice model in which Macf1 was specifically deleted in osteoblasts by Cre‐*loxP* recombination system. Here, we showed that deficiency of Macf1 decreased bone mass, deteriorated bone microarchitecture and impaired bone strength. In addition, we found that knockout of Macf1 inhibited the differentiation of primary osteoblasts through Bmp2/Smad/Runx2 pathway. Our studies revealed novel insights into the function and mechanism of Macf1 in bone formation. Moreover, we provided a new mice model for in vivo function research of Macf1 and targeted therapy research of osteoporosis.

## 2 MATERIALS AND METHODS

### 2.1 Generation of *Macf1^f/f^Osx*‐Cre mice

*Osterix*‐Cre (*Osx*‐Cre) and *Macf1^flox/flox^* (*Macf1^f/f^*) C57BL/6 mouse lines used in the current study have been previously described.^6^, ^25^ *Osx*‐Cre mice were crossed with *Macf1^f/f^* mice, and their progeny were bred to obtain osteoblast‐specific conditional knockout mice (*Macf1^f/f^Osx*‐Cre). *Macf1^f/f^* mice were used as control.

The genotypes were determined by PCR amplification of genomic DNA isolated from the toes or tails of newborn mice. PCR was conducted in an BIO‐GENER GE4852T thermocycler (BIO‐GENER) with an initial denaturation at 95°C for 5 minutes; then 35 cycles of 95°C for 30 seconds, 55°C for 30 seconds, 72°C for 30 seconds; and a final round at 72°C for10 minutes. 1% agarose gels (HydraGene) stained with 0.1% GoldView (Hat Biotechnology) were used to visualize the PCR products. Sequences of the primers used for genotyping have been listed in Table 1.

**Table 1:** Primer sequences for genotyping

	Sequence	Product size
*Macf1*‐*Flox*	F‐5′‐AAAGAAACGGAAATAGGCC‐3′	*Macf1*‐*Flox*‐WT: ~700bp *Macf1*‐*Flox*‐Mut: ~750bp
	R‐5′‐GCAGCTTAATTCTGCAAATTC‐3′	
*Osx*‐Cre‐WT	F‐5′‐TACCAGAAGCGACCACTTGAGC‐3′	*Osx*‐Cre‐WT: 263bp
	R‐5′‐CGCCAAGAGAGCCTGGCAAG‐3′	
*Osx*‐Cre‐Mut	F‐5′‐TACCAGAAGCGACCACTTGAGC‐3′	*Osx*‐Cre‐Mut: 445bp
	R‐5′‐GCACACAGACAGGAGCATCTTC‐3′	

All mice were maintained on a 12 hours light/dark cycle with free access to water and standard laboratory food. All animal experiments were performed in accordance with the Guiding Principles for the Care and Use of Laboratory Animals, and all experimental procedures were approved by the Institutional Experimental Animal Committee of Northwestern Polytechnical University (Xi'an, China).

### 2.2 Primary osteoblasts isolation and osteogenic differentiation

Primary osteoblasts were isolated from calvaria of mice on postnatal day 1 as previously described.^26^ Briefly, calvaria was dissected and sequentially digested by digestion solution containing 0.1% type II collagenase (Invitrogen, CA, USA) and 0.2% neutral protease (Kehao) in hanks’ balanced salt solution for 15 minutes at 37°C. The cells isolated from the first and second digestions were discarded, and cells obtained from later four digestions were cultured in α‐MEM (Gibco) supplemented with 10% FBS (Corning), 1% L‐glutamine (Sigma), 100 U/mL penicillin (Amresco) and 100 μg/mL streptomycin (Amresco).

To induce osteogenic differentiation, the primary osteoblasts were cultured in osteogenic medium in α‐MEM (Gibco) supplemented with 10% FBS (ExCell), 1% L‐glutamine (Sigma), 50 μg/mL ascorbic acid (Sigma), 10 mmol/L β‐glycerophosphate (Sigma), 100 U/mL penicillin (Amresco) and 100 μg/mL streptomycin (Amresco). 100 ng/mL recombinant human BMP2 (rhBMP2, PeproTech) was added to osteogenic medium when needed. Then, cells were subjected to Alkaline phosphatase staining at 7 days after differentiation and Alizarin red S staining at 14 days after differentiation.

### 2.3 Alizarin red and Alcian blue staining

Skeletal structures of mice at postnatal day 0 were analysed by whole‐mount Alizarin red/Alcian blue staining. All mice were skinned and eviscerated, and fixed in 95% ethanol overnight. Fat tissues were removed with acetone at room temperature. Cartilage tissue of mice was stained with Alcian blue (Sigma) for 24 hours. After rinsed in 70% ethanol for 8 hours, soft tissues were removed with 1% KOH. Bones were counterstained with Alizarin red (Sigma) overnight and subsequently cleared with 1% KOH/20% glycerol for 2 days or more at 4°C. Samples were stored in glycerol:ethanol (1:1) until imaging. The width of cranial suture, mineralized region rate of 9th rib and mineralized region rate of femur were measured by Image J analysis software (Image J).

For Alizarin red S (ARS) staining of cells, osteoblasts after differentiation in 24‐well plate were washed with PBS, fixed with 4% Paraformaldehyde (PFA) at room temperature for 15 minutes and stained with 0.5% alizarin red staining solution (pH = 4.0) (Sigma) for 20 minutes at room temperature. Then, the cells were washed with tap water for 3‐5 times. The mineralized nodules were scanned by a CanoScan 9000F Mark II scanner (Canon).

### 2.4 Alkaline phosphatase staining

Alkaline phosphatase (ALP) staining was performed with a BCIP/NBT ALP colour development kit (Beyotime). Briefly, cells were rinsed 3 times with PBS and fixed with 4% PFA. Then, the osteoblasts were incubated with 500 μL/well of BCIP/NBT substrate for about 2 hours in the dark. After washing with PBS, the ALP active cells were imaged.

### 2.5 Western blot analysis

Tibia, liver, spleen and heart of the male mice were immediately separated and pulverized in a cooled mortar with liquid nitrogen. The bone marrow in tibia was flushed out before pulverization. Primary osteoblasts were washed with ice‐cold PBS before protein extraction. Whole‐cell lysates for Western blot were extracted using RIPA Lysis buffer (Beyotime) with protease and phosphatase inhibitor cocktails (Calbiochem). The concentration of protein was analysed by BCA protein assay. Protein lysates were separated using 10% SDS‐PAGE at 120V for 1 hour and then electro‐transferred to nitrocellulose membranes at 100V for 2 hours. The membranes were blocked with 5% non‐fat milk in TBST and then incubated with antibodies against Macf1 (1:500, Abcam), Alp (1:1000, Santa Cruz Biotech), Col1 (1:1000, Cusabio Technology), Bmp2 (1:1000, Servicebio Technology), p‐Smad1/5/9 (1:1000, Cell Signalling Technology), Smad1 (1:1000, Cusabio Technology), Runx2 (1:500, Servicebio Technology) and Gapdh (1:2000, Servicebio Technology) overnight at 4°C, followed by incubation with corresponding secondary antibodies for 2 hours at room temperature. Protein bands were facilitated by Western Bright ECL Spray (Advansta San Jose) and visualized by a T5200 Multi chemiluminescence detection system (Tanon). Densitometric quantification of the bands was performed with Image J analysis software (Image J).

### 2.6 Quantitative real‐time PCR (qPCR)

Total RNA was extracted from primary osteoblasts or pulverized tissues by TRIzol reagent (Invitrogen) according to the manufacturer's instructions. 1 μg of total RNA was reverse‐transcribed into cDNA by PrimeScript RT reagen Kit (TaKaRa). Real‐time PCR assay for mRNA detection was performed by SYBR qPCR Master (TaKaRa) using fluorescence quantitative detection system (Line‐Gene 9600, BIOER) with an initial denaturation at 95°C for 30 seconds, followed by 45 cycles at 95°C for 10 seconds, 60°C for 30 seconds and 72°C for 5 seconds. Primers used for qPCR were listed in Table 2. *Gapdh* was used as control for mRNA analysis.

**Table 2:** Primer sequences for qPCR

Target	Sequence	Accession No.
*Macf1*	F‐5′‐GAAAACATTCACCAAGTGGGTCAAC‐3′	NM_001199137.1
	R‐5′‐TGTCCATCCCGAAGGTCTTCATAG‐3′	
*Alp*	F‐5′‐GTTGCCAAGCTGGGAAGAACAC‐3′	NM_007431.1
	R‐5′‐CCCACCCCGCTATTCCAAAC‐3′	
*Col1*	F‐5′‐GAAGGCAACAGTCGATTCACC‐3′	NM_007742.3
	R‐5′‐GACTGTCTTGCCCCAAGTTCC‐3′	
*Runx2*	F‐5′‐CGCCCCTCCCTGAACTCT‐3′	NM_001145920.1
	R‐5′‐TGCCTGCCTGGGATCTGTA‐3′	
*Gapdh*	F‐5′‐TGCACCACCAACTGCTTAG‐3′	NM_008084.2
	R‐5′‐GGATGCAGGGATGATGTTC‐3′	

### 2.7 Dual energy X‐ray absorptiometry (DXA)

After anesthetized with 15 μg/mL pentobarbital sodium, mice were placed on the specimen tray of DXA body composition analysis system (InAlyzer, Medikors) in a prone or side position for whole body scanning. Radiographic images and related parameters of different bone regions were obtained using InAlyzer Dual X‐ray Digital Imaging Software (InAlyzer, Medikors).

### 2.8 Micro‐Computed Tomography (micro‐CT)

Micro‐Computed Tomography analysis was performed as previously described.^27^ Briefly, the distal femurs were scanned ex vivo by a micro‐CT system (viva CT40, Scanco Medical) with a voxel size of 10 μm and an integration time of 200 ms A total of 426 slices were scanned along the femur diaphysis beginning at the growth plate and extending proximally. Eighty continuous slices starting at 0.1 mm from the most proximal side of the growth plate were selected for analysis. The trabecular bone from selected slices was segmented for three‐dimensional reconstruction (Sigma = 0.8, Supports = 1 and Threshold = 200) and morphometric parameters calculation. The values for bone mineral density (BMD), bone volume fraction (bone volume per tissue volume, BV/TV), trabecular thickness (Tb.Th.), trabecular number (Tb.N.), trabecular separation (Tb.Sp) and structure model index (SMI) were quantified to evaluate trabecular microarchitecture.

### 2.9 Bone mechanical properties

The biomechanical strength of femurs was analysed by three‐point bending test performed on an Instron 5943 universal test machine (Instron). The span between the lower supports was set to 10 mm. The upper contact point was aligned at the midpoint of the lower supports. The upper actuator moved at a rate of 1.5 mm/min till the specimen was failed, and the load‐displacement curves were received. The inner and outer diameters of loaded bones were measured by a 3D digital microscope (Hirox KH‐8700). Other parameters including fracture energy, young's modulus, peak bending stress and strain were determined using standard algorithms as previously described.^28^

### 2.10 Double calcein labelling

For new bone formation rate analysis, mice were intraperitoneally injected with calcein green (Sigma) (20 mg/kg bodyweight) in a time sequence of 10 days and 3 days before sacrifice. Femurs were fixed with 4% PFA, dehydrated in graded ethanol and embedded in methyl methacrylate (Sigma). 15‐μm sections were obtained using Leica SM2500E microtome (Leica Microsystems). Bone dynamic histomorphometric analyses for mineral apposition rate (MAR) were performed by Image J analysis software (Image J) using images captured from a Leica Q500MC fluorescence microscope (Leica).

### 2.11 Histology and immunohistochemistry

Femurs were harvested, fixed with 4% PFA and decalcificated with 10% EDTA for about 1 month. After dehydrated, specimens were embedded in paraffin. 3‐μm sections were prepared for haematoxylin and eosin (HE) staining and Immunohistochemistry (IHC) staining. All staining procedures were performed according to the standard procedures. IHC was performed using primary antibodies against Ocn (Servicebio Technology), Bmp2 (Servicebio Technology) and Runx2 (Servicebio Technology) at 1:200 dilution, followed by HRP‐conjugated secondary antibody (Invitrogen). Then, signal was detected by 3, 3′‐diaminobenzidine tetrahydrochloride (DAB) kit (Invitrogen).

### 2.12 Statistical analyses

Statistical analysis was performed using Graph Pad PRISM 5.0. All results are expressed as mean ± SD. Unpaired Student's *t* tests were used to compare data between two *Macf1^f/f^* and *Macf1^f/f^Osx*‐Cre mice groups. For all experiments, significance was defined as *P < .05, **P < .01 and ***P < .001.

## 3 RESULTS

### 3.1 Generation of Macf11 conditional knockout mice

**Figure 1:**
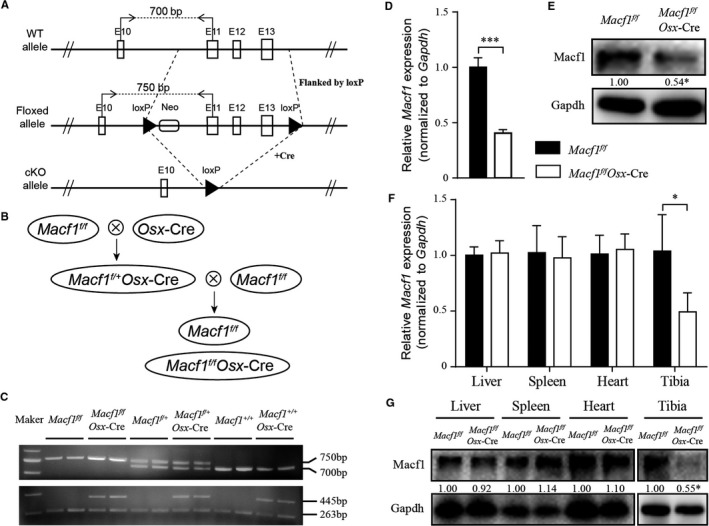
Generation of Macf1 conditional knockout mice. (A) Schematic illustration of the wild‐type allele (WT allele), floxed *Macf1* allele before (Floxed allele) and after (cKO allele) deletion of the *loxP* cassette containing exon 11‐13 by Cre‐mediated recombination. “//” indicated that all the introns and exons were omitted before exon 10 and after exon 13. (B) Breeding scheme used to generate *Macf1^f/f^Osx*‐Cre mice. *Macf1^f/f^* mice were used as control. (C) PCR analysis of genomic DNA isolated from the toes or tails of progeny mice of different genotypes. (D, E) The mRNA and protein expression of Macf1 in primary osteoblasts obtained from calvarial of newborn *Macf1^f/f^* and *Macf1^f/f^Osx*‐Cre mice were measured by qPCR (D) and Western blot (E); densitometric analysis of Western blotting images are indicated as numbers below the blots. (F and G) The mRNA and protein expression of Macf1 in liver, spleen, heart and tibia of *Macf1^f/f^* and *Macf1^f/f^Osx*‐Cre mice were measured by qPCR (F) and Western blot (G); densitometric analysis of Western blotting images is indicated as numbers below the blots. Data are means of triplicate experiments ± SEM. *P < .05, ***P < .001

To investigate the role of Macf1 in osteogenesis, Mice with floxed alleles for *Macf1* with *loxP* sites flanking from exons 11 to 13 (*Macf1^f/f^*) were crossed with *Osx*‐Cre mice to generate osteoblast‐specific conditional knockout mice (*Macf1^f/f^Osx*‐Cre) (Figure 1A). The breeding strategy produced both *Macf1^f/f^Osx*‐Cre mice and *Macf1^f/f^* control mice were shown in Figure 1B. PCR analysis was performed to identify the genotype of offspring in the breeding processes (Figure 1C). In addition, qPCR and Western blot results showed that the expression of Macf1 in primary osteoblasts was significantly decreased in *Macf1^f/f^Osx*‐Cre mice compared with that of littermate *Macf1^f/f^* mice (Figure 1D,1). The specific deletion of Macf1 in bone tissue was confirmed by comparing with other tissues by qPCR and Western blot (Figure 1F, G). Moreover, the *Macf1^f/f^Osx*‐Cre mice were viable and phenotypically normal, and showed no obvious variances in behaviour compared with normal mice.

### 3.2 Deficiency of Macf1 delayed bone ossification and decreased bone mass

**Figure 2:**
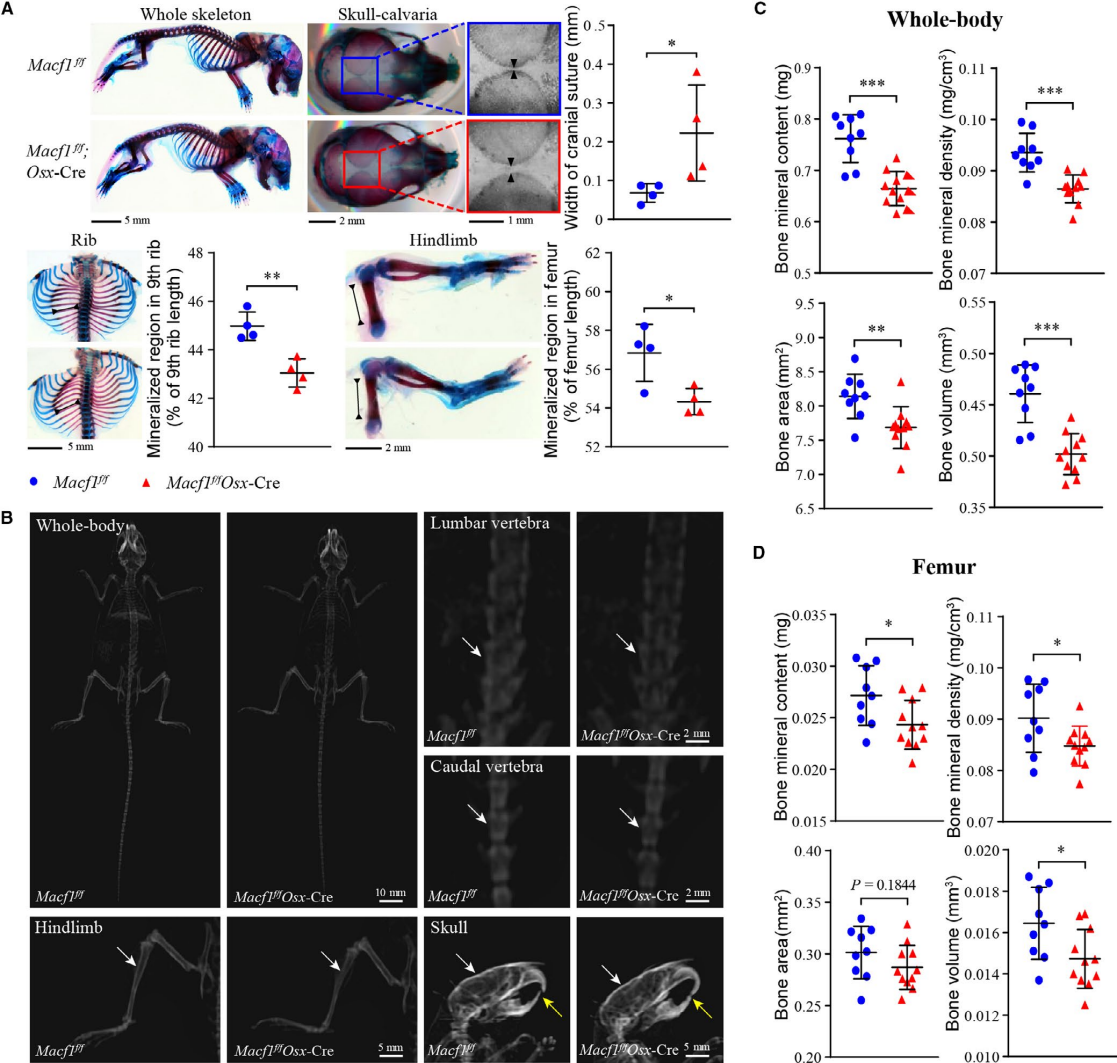
Deficiency of Macf1 delayed bone ossification and decreased bone mass. (A) Alizarin red and Alcian blue staining of whole‐mount skeletal of newborn Macf1*^f/f^* and Macf1*^f/f^*Osx‐Cre mice (n = 4 per group). Bone ossification of skull, rib and hindlimb was quantified by width of cranial suture, mineralized region rate of 9th rib and mineralized region rate of femur, respectively. (B) DXA analysis of hindlimb, lumbar vertebra, caudal vertebra and skull from 3‐month‐old Macf1*^f/f^* and Macf1*^f/f^*Osx‐Cre mice. The radiodensity of hindlimb, lumbar vertebra, caudal vertebra and skull was indicated by white arrows. (C, D) Quantification of bone mineral content, bone mineral density, bone area and bone volume in whole body (C) and femur (D) from Macf1*^f/f^* and Macf1*^f/f^*Osx‐Cre mice (n = 9 for Macf1*^f/f^*, n = 11 for Macf1*^f/f^*Osx‐Cre). Data are presented as means ± SEM. *P < .05, **P < .01 and ***P < .001

To determine the effects of Macf1 deficiency on bone ossification during skeletal development, skeletons of newborn *Macf1^f/f^* and *Macf1^f/f^Osx*‐Cre mice (P0) were stained with Alcian blue and Alizarin red. The results showed that the ossification of skull, rib and hindlimb was delayed in *Macf1^f/f^Osx*‐Cre mice compared with that of *Macf1^f/f^* mice (Figure 2A). The bone mass of 3‐month‐old *Macf1^f/f^* and *Macf1^f/f^Osx*‐Cre mice was measured by DXA analysis. Radiographs showed a significantly decreased bone mass in hindlimb, lumbar vertebra, caudal vertebra and skull (Figure 2B, indicated by white arrows). Quantification showed that, compared with that of *Macf1^f/f^* mice, the bone mineral density of the whole body and femur was reduced by 7.6% and 6.0% in *Macf1^f/f^Osx*‐Cre mice, respectively (Figure 2C,2). Bone mineral content, bone area and bone volume were also reduced in *Macf1^f/f^Osx*‐Cre mice compared with control (Figure 2C,2). Besides, we also found that the incisors displayed a white and opaque appearance in *Macf1^f/f^Osx*‐Cre mice, which were in clear contrast to the yellow‐brown and transparent glossy appearance of normal incisors (data were not shown). DXA images showed blunter and shorter mandibular incisors in Macf1 deletion mice (Figure 2B, indicated by yellow arrows), implying that the deficiency of Macf1 might induce the formation of softer enamel. These results indicated that deletion of Macf1 in osteoblasts delayed ossification during bone development and decreased bone mass in adult mice.

### 3.3 Deficiency of Macf1 impaired bone microarchitecture

**Figure 3:**
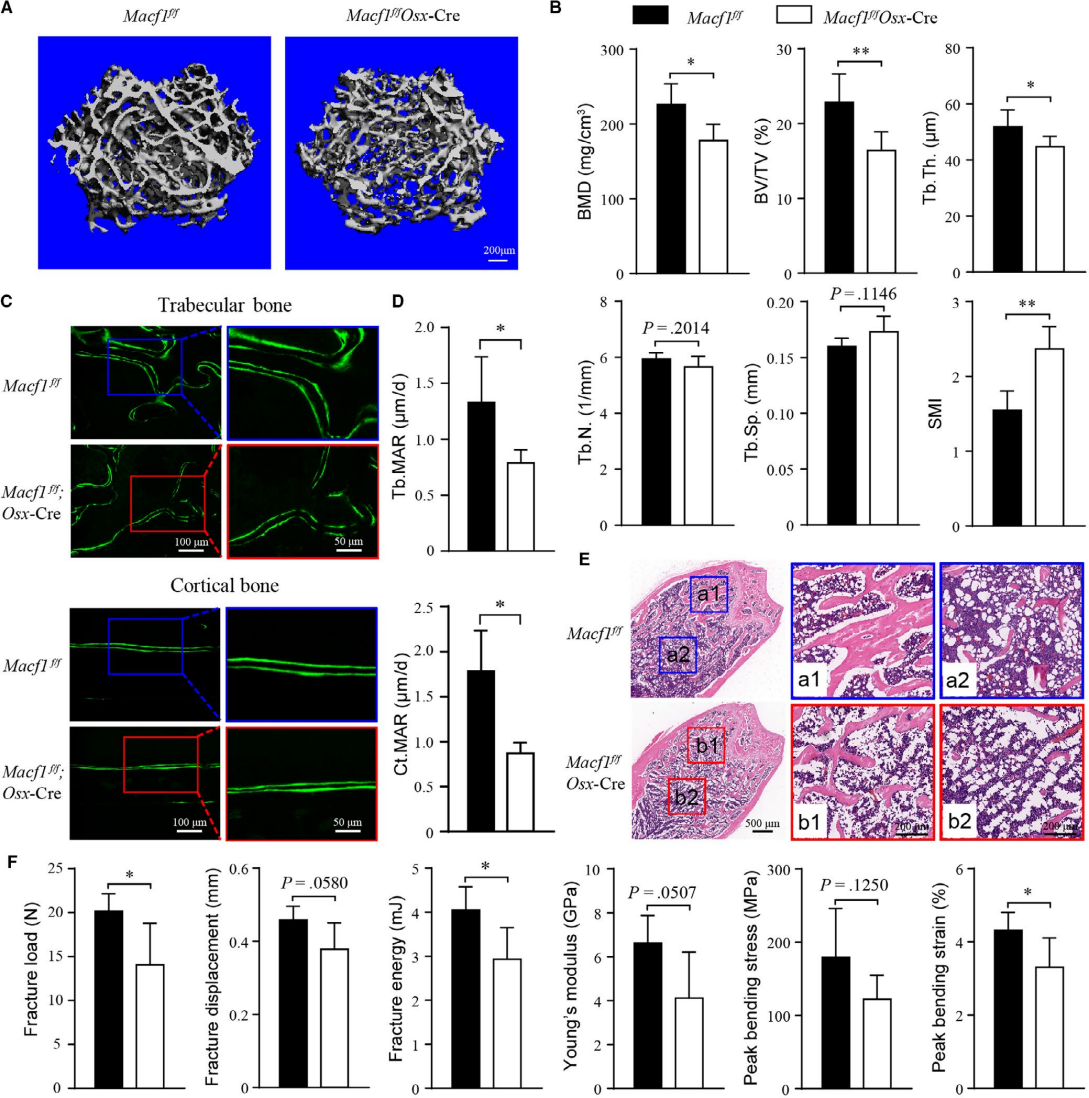
Deficiency of Macf1 impaired bone formation and microoarchitecture. (A) Representative micro‐CT images of femur from 3‐month‐old *Macf1^f/f^* and *Macf1^f/f^Osx*‐Cre mice. (B) Micro‐CT analysis of femur from *Macf1^f/f^* and *Macf1^f/f^Osx*‐Cre mice. BMD, bone mineral density; BV/TV, bone volume per tissue volume; Tb.Th., trabecular thickness; Tb.N., trabecular number; Tb.Sp, trabecular separation; and SMI, structure model index (n = 4 for *Macf1^f/f^*, n = 7 for *Macf1^f/f^Osx*‐Cre). (C) Representative fluorescent images of calcein double labelling of femur from 3‐month‐old *Macf1^f/f^* and *Macf1^f/f^Osx*‐Cre mice showing decreased bone mineralization in trabecular and cortical bone. (D) Quantification of mineral apposition rate in femur of 3‐month‐old *Macf1^f/f^* and *Macf1^f/f^Osx*‐Cre mice. Tb.MAR, trabecular mineral apposition rate (n = 5 for *Macf1^f/f^*, n = 6 for *Macf1^f/f^Osx*‐Cre) and Ct.MAR, cortical mineral apposition rate (n = 4 for *Macf1^f/f^*, n = 3 for *Macf1^f/f^Osx*‐Cre). (E) Representative HE staining images of femur from 3‐month‐old *Macf1^f/f^* and *Macf1^f/f^Osx*‐Cre mice. The boxed area was magnified to show the trabecular bone (a1 and b1) and accumulated adipocytes (a2 and b2) in bone marrow. (F) Biomechanical properties of fracture load, fracture displacement, fracture energy, Young's modulus, peak bending stress and peak bending strain in femur of 3‐month‐old *Macf1^f/f^* and *Macf1^f/f^Osx*‐Cre mice femur (n = 5 per group). Data are presented as means ± SEM. *P < .05, **P < .01 and ***P < .001

Micro‐Computed Tomography was used to determine the effect of Macf1 on the structural parameters of trabecular bone from 3‐month‐old *Macf1^f/f^* and *Macf1^f/f^Osx*‐Cre mice. The micro‐CT images of trabecular architecture of distal femurs showed that the *Macf1^f/f^Osx*‐Cre mice displayed a lower bone mass than *Macf1^f/f^* mice (Figure 3A). Consistently, quantification of structural parameters of the trabecular bone under the growth plate in the distal femur indicated a significant reduction of bone mineral density (BMD) and bone volume over the total volume of the tissue (BV/TV) in *Macf1^f/f^Osx*‐Cre mice relative to the *Macf1^f/f^* controls. Likewise, the decrease of trabecular connectivity was confirmed by a significant decrease in trabecular thickness (Tb.Th.) and increase in structure model index (SMI) in *Macf1^f/f^Osx*‐Cre mice. Trabecular number (Tb.N.) and trabecular spacing (Tb.Sp) changed a little but were not significant in *Macf1^f/f^Osx*‐Cre mice compared with control mice (Figure 3B).

Furthermore, we determined whether the reduction of bone mass in *Macf1^f/f^Osx*‐Cre mice was due to decreased bone formation. Bone histomorphometric analysis was performed in sections of femur from 3‐month‐old mice. Double labelling of calcein was used to analyse the dynamic bone marker mineral apposition rate (MAR) at the distal femur. The distance between two labelled mineralization fronts was reduced in trabecular and cortical bone form *Macf1^f/f^Osx*‐Cre mice compared with *Macf1^f/f^* mice (Figure 3C). Histomorphometric measurements showed that the MAR of trabecular and cortical bone in *Macf1^f/f^Osx*‐Cre mice was 40.7% and 51.3% lower than that in littermate *Macf1^f/f^* mice, respectively (Figure 3D). Additionally, HE staining showed that trabecular bones were less developed in *Macf1^f/f^Osx*‐Cre than in littermate *Macf1^f/f^* mice (Figure 3E). Meanwhile, we unexpectedly observed an increased number of adipocyte‐like vacuoles in the bone marrow of *Macf1^f/f^Osx*‐Cre mice (Figure 3E). These results suggested that Macf1 was required for trabecular bone formation and normal bone microarchitecture.

### 3.4 Deficiency of Macf1 impaired bone biomechanical properties

The effect of bone quality on biomechanical properties is determined by the material composition and microarchitecture of bone.^29^ Based on the above findings, the biomechanical strength of the femurs from 3‐month‐old *Macf1^f/f^* and *Macf1^f/f^Osx*‐Cre mice was analysed by three‐point bending test. As shown in Figure 3F, the biomechanical properties of *Macf1^f/f^Osx*‐Cre mice were dramatically reduced compared with *Macf1^f/f^* mice. The fracture load, displacement, energy, young's modulus, peak bending stress and strain were all reduced in *Macf1^f/f^Osx*‐Cre mice by 30.4%, 17.5%, 27.5%, 37.8%, 31.9% and 23.6%, respectively. Thus, these data indicated that deficiency of Macf1 reduced the biomechanical strength of femur.

### 3.5 Osteoblasts specific deletion of Macf1 inhibited osteogenic differentiation

**Figure 4:**
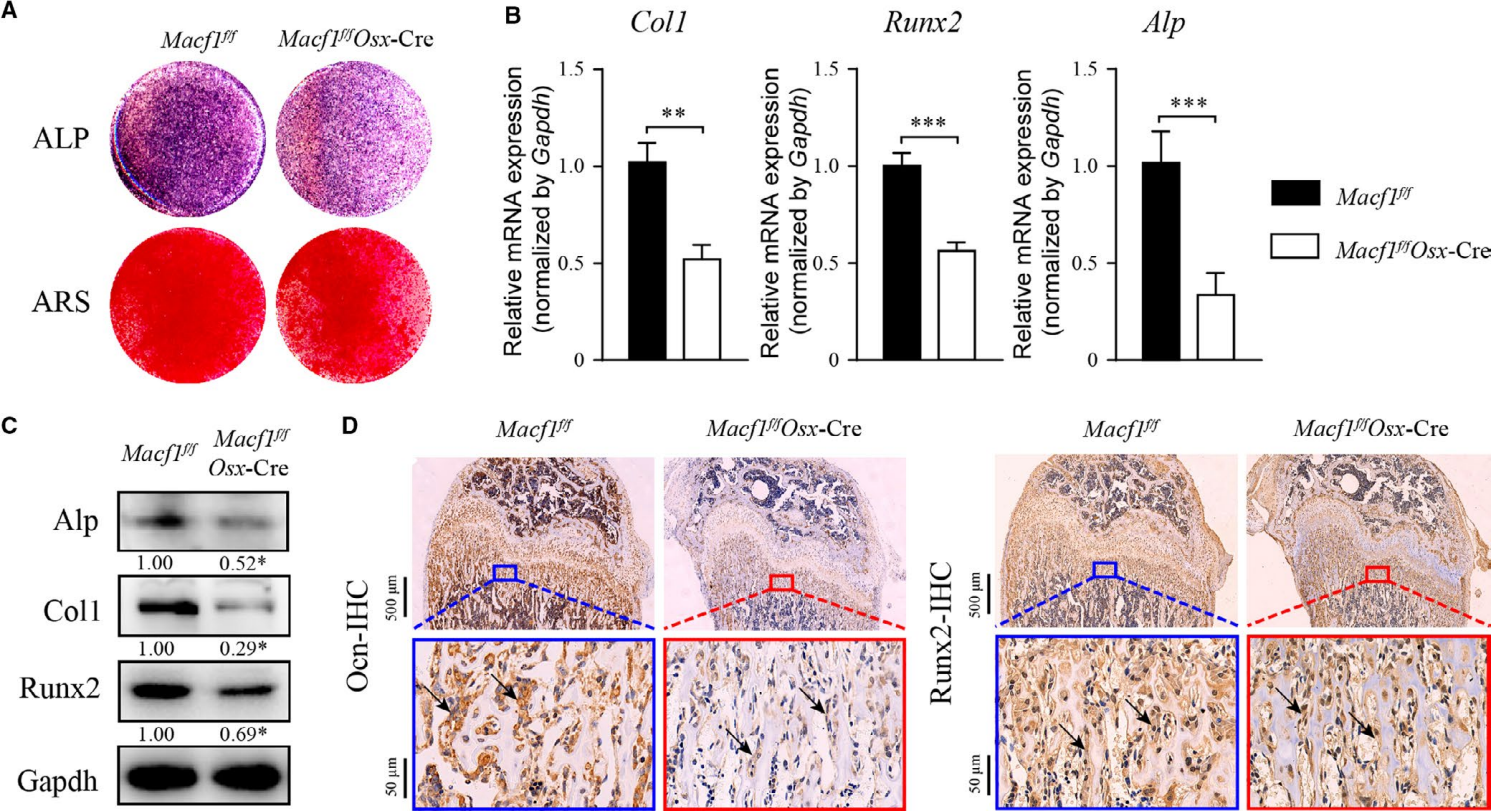
Deficiency of Macf1 inhibited osteoblast differentiation. (A) ALP staining at day 7 and ARS staining at day 14 of osteogenic‐induced primary osteoblasts from calvaria of newborn *Macf1^f/f^* and *Macf1^f/f^Osx*‐Cre mice. (B, C) qPCR (B) and Western blot (C) analysis of the of Runx2, Col1 and Alp expression in 7 days osteogenic‐induced primary osteoblasts from calvarial of *Macf1^f/f^* and *Macf1^f/f^Osx*‐Cre mice. Densitometric analysis of Western blotting images is indicated as numbers below the blots. (D) Representative IHC images of Ocn and Runx2 in femur from 4‐week‐old *Macf1^f/f^* and *Macf1^f/f^Osx*‐Cre mice (indicated by black arrows). Data are means of triplicate experiments ± SEM. *P < .05, **P < .01 and ***P < .001

To further determine whether the deletion of Macf1 inhibited the differentiation of osteoblast in vitro, primary osteoblasts were isolated from the calvaria of newborn *Macf1^f/f^* and *Macf1^f/f^Osx*‐Cre mice and cultured with osteogenic medium. After 7 and 14 days of differentiation, the ALP activity and numbers of mineralized nodules of osteoblasts were measured by ALP staining and ARS staining, respectively. The results showed that deletion of Macf1 inhibited ALP activity and decreased mineralization of primary osteoblasts (Figure 4A). Next, the expressions of osteogenic markers were measured by qPCR and Western blot. The results showed that the mRNA and protein expression of Alp, Col1 and Runx2 were decreased in primary osteoblasts of *Macf1^f/f^Osx*‐Cre mice after 7 days induction (Figure 4B,4). In addition, IHC assay showed that the expression of Runx2 and Ocn was decreased in the femur of 3‐month‐old *Macf1^f/f^Osx*‐Cre mice (Figure 4D). Taken together, these data suggested that osteoblast‐specific deletion of Macf1 inhibited the differentiation of primary osteoblasts.

### 3.6 Macf1 deficiency inhibited osteoblast differentiation via Bmp2/Smad/Runx2 pathway

**Figure 5:**
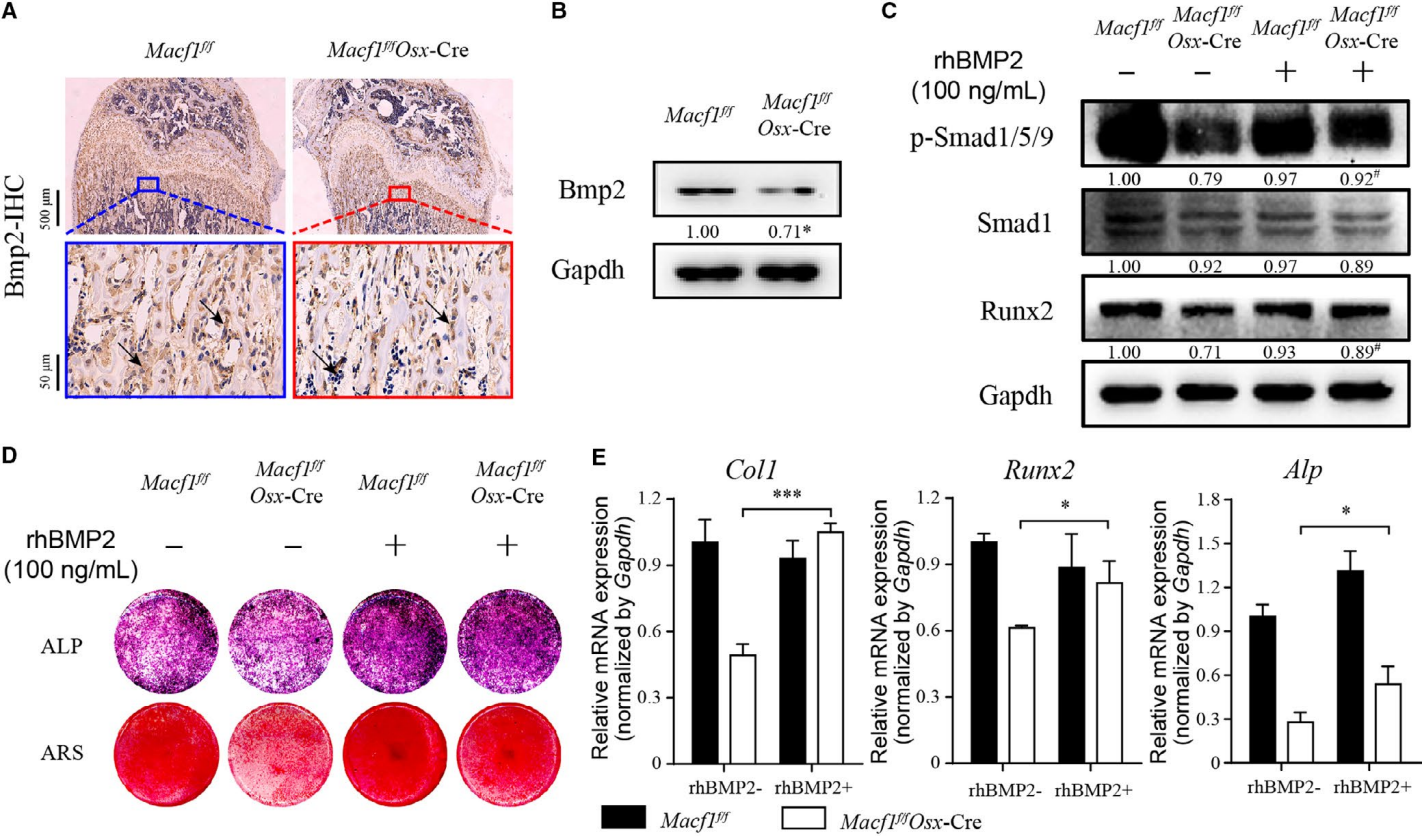
Deficiency of Macf1 inhibited osteogenic differentiation of primary osteoblasts through Bmp2/Smad/Runx2 pathway. (A) Representative IHC images of Bmp2 in femur from 4‐week‐old *Macf1^f/f^* and *Macf1^f/f^Osx*‐Cre mice (indicated by black arrows). (B) Western blot analysis of Bmp2 level in 7 days osteogenic‐induced primary osteoblasts from calvarial of *Macf1^f/f^* and *Macf1^f/f^Osx*‐Cre mice. Densitometric analysis of Western blotting images is indicated as numbers below the blots. (C) Western blot analysis of p‐Smad1/5/9, Smad1 and Runx2 level of 7 days osteogenic‐induced primary osteoblasts from calvarial of *Macf1^f/f^* and *Macf1^f/f^Osx*‐Cre mice treated with or without rhBMP2. Densitometric analysis of Western blotting images is indicated as numbers below the blots. (D) ALP and ARS staining of osteogenic‐induced primary osteoblasts from calvarial of *Macf1^f/f^* and *Macf1^f/f^Osx*‐Cre mice treated with or without rhBMP2. (E) qPCR analysis of the expressions of *Runx2*, *Col1* and *Alp* in 7 days osteogenic‐induced primary osteoblasts from calvarial of *Macf1^f/f^* and *Macf1^f/f^Osx*‐Cre mice treated with or without rhBMP2. Data are means of triplicate experiments ± SEM. *P < .05, **P < .01, ***P < .001, ^#^ *P* < .05 vs *Macf1^f/f^Osx*‐Cre without rhBMP2 treatment

To clarify whether Macf1 could regulate osteogenesis by modulating the Bmp2/Smad/Runx2 signalling pathway, we first analysed the expression of Bmp2 in femur of *Macf1^f/f^Osx*‐Cre mice. As expected, the IHC assay showed that the expression of Bmp2 was inhibited in the femur of *Macf1^f/f^Osx*‐Cre mice compared with littermate *Macf1^f/f^* mice (Figure 5A). What is more, the protein expression of Bmp2 was inhibited in primary osteoblasts of *Macf1^f/f^Osx*‐Cre mice (Figure 5B). We next treated the primary osteoblasts from *Macf1^f/f^* and *Macf1^f/f^Osx*‐Cre mice with 100 ng/mL rhBMP2, respectively. After 7 days of induction, the protein expression of p‐Smad1/5/9 and Runx2 in primary osteoblasts was inhibited by deletion of Macf1, and this inhibition was rescued by exogenous rhBMP2 (Figure 5C). ALP and ARS staining results showed that the differentiation capability was significantly recovered in primary osteoblasts from *Macf1^f/f^Osx*‐Cre mice by rhBMP2 (Figure 5D). Expressions of osteogenic marker genes in osteoblasts were also recovered after rhBMP2 treatment (Figure 5E). These results demonstrated that deficiency of Macf1 inhibited osteogenic differentiation of primary osteoblasts via Bmp2/Smad/Runx2 pathway.

## 4 DISCUSSION

In the present study, we constructed an osteoblast‐specific *Osterix* promoter‐driven Macf1 conditional knockout mice model utilizing Cre‐*loxP* recombination system. The mice model was proved successful by detecting the mRNA and protein expression levels of Macf1 in primary osteoblasts and bone tissues. Our data demonstrated that deficiency of Macf1 in osteoblast reduced bone formation and osteoblast differentiation by affecting the Bmp2/Smad/Runx2 pathway. The research provides a mice model to further study the functions of Macf1 in vivo.

Previous studies have revealed critical roles of Macf1 in mammalian skin, intestine, heart, brain, retina and skeletal muscle.^3^, ^7^, ^10^, ^11^, ^12^, ^30^, ^31^, ^32^, ^33^ However, the function and mechanism of Macf1 in bone formation in vivo are still unclear. Chen et al have reported that global deletion of Macf1 induces embryonic lethality.^5^ In the current study, the Cre‐*loxP* recombination system was used to establish osteoblast‐specific *Osterix* promoter‐driven Macf1 conditional knockout mice. Macf1 is significantly decreased in primary osteoblast and bone tissue, indicating that the model is successfully constructed.

Previous studies have shown important roles of Macf1 in brain, muscle and lung development, and that Macf1 is crucial in maintaining normal tissue structure and functions of muscle and neural.^12^, ^13^, ^31^, ^32^, ^34^ In this study, we showed that deletion of Macf1 in osteoblasts delayed bone ossification of newborn mice and decreased the bone mass of adult mice. Furthermore, we found that deficiency of Macf1 induced a reduction of bone formation rate, a deterioration of bone microarchitecture and a weaker strength of bone, indicating a potential function of Macf1 in bone development and formation. In addition, previous studies have demonstrated that osteoblasts and adipocytes in bone marrow share a common progenitor, and the dynamic balance between them can influence the density and function of bone.^35^ In this study, histological staining of Macf1 deficiency femur displayed an increased accumulation of adipocyte ghosts in situ (Figure 3E). This phenotype is similar to the observations following the β‐catenin and Cbfβ deletion in Osx‐expressing cells.^36^, ^37^ It would be interesting to examine the function and mechanism of Macf1 on bone marrow adiposity in the future.

Osteoblasts are uniquely responsible for bone formation.^38^ Our previous studies showed that knockdown of Macf1 inhibited the differentiation of MC3T3‐E1 osteoblastic cell line in vitro.^17^ In this study, we found that primary osteoblasts isolated from *Macf1^f/f^Osx*‐Cre mice showed decreased ALP activity and mineralization after osteogenic differentiation. The expression of osteogenic marker genes was reduced in both primary osteoblasts and bone tissues of *Macf1^f/f^Osx*‐Cre mice. Taken together, these studies indicated that Macf1 played positive role in the differentiation of primary osteoblasts, which was consistent with the results of our previous studies in MC3T3‐E1 cell line.

Chen et al and our groups have previously reported that Macf1 participates in regulation of wnt/β‐catenin signalling.^5^, ^17^ Zhang et al have demonstrated that Wnt/β‐catenin signalling pathway is an upstream activator of Bmp2 expression in osteoblasts.^24^ Bmp2/Smad/Runx2 signalling pathway is a crucial regulator of osteogenic differentiation.^21^ In this pathway, Bmp2 induces phosphorylation of Smad1/5/9 and then initiates Runx2 transcription factor.^22^ In our study, we found that the expression of Bmp2 was decreased in both bone tissue and primary osteoblasts after deletion of Macf1. Deficiency of Macf1 inhibited the protein expression of Smad1/5/9 and Runx2 in primary osteoblasts. After treated with recombinant human BMP2 in primary osteoblasts from *Macf1^f/f^* and *Macf1^f/f^Osx*‐Cre mice, the down‐regulation of the Smads and Runx2 in Macf1‐delected osteoblasts was significantly recovered. In addition, the differentiation ability of Macf1 knockout osteoblasts was rescued accordingly. Together with the present results, we concluded that Macf1 could regulate osteoblast differentiation through Bmp2/Smad/Runx2 signalling pathway.

In summary, we successfully generated an osteoblast‐specific Macf1 conditional knockout mice model using Cre‐*loxP* system. Our data demonstrated that deletion of Macf1 decreased bone mass, deteriorated bone microarchitecture and impaired bone strength. Macf1 could affect the differentiation and mineralization of osteoblasts through Bmp2/Smad/Runx2 signalling pathway. Our study not only reveals a novel role and mechanism of Macf1 in bone formation, but also provides a mice model to further study the functions of Macf1 in vivo.

## CONFLICT OF INTEREST

The authors declare that they have no conflicts of interest to disclose.

## AUTHOR CONTRIBUTIONS

WX Qiu, X. Lin, F. Zhao and AR Qian studied design. WX Qiu, X. Lin, XL Ma and AR Qian contributed to development of methodology. WX Qiu, F. Zhao, DJ Li, R. Zhang, P. Wang, KW Zhang, ZH Chen and YY Xiao contributed to acquisition of data. WX Qiu, X. Lin, XY Wu, F. Zhao, DJ Li, ZP Miao and K. Dang involved in analysis and interpretation of data. WX Qiu, X. Lin and AR Qian wrote, reviewed and/or revised the manuscript. X. Lin and AR Qian studied supervision and all authors read and approved the final manuscript.

## Data Availability

The data that support the findings of this study are available from the corresponding author upon reasonable request.
